# Esophageal perforation by tilapia fish bone ingestion – A case report

**DOI:** 10.1016/j.ijscr.2020.04.054

**Published:** 2020-05-11

**Authors:** Radisnay Guzman Lambert, Seth Kwadjo Angmorterh, Manuel Betancourt Benjamin, Mariuska Rodriguez Gonzalez, Sonia Aboagye, Eric Kwasi Ofori

**Affiliations:** a Department of Surgery, School of Medicine, University of Health and Allied Sciences (UHAS), Ho, Ghana; b Department of Medical Imaging, School of Allied Health Sciences, University of Health and Allied Sciences (UHAS), Ho, Ghana; c Department of Radiology, School of Medicine, University of Health and Allied Sciences (UHAS), Ho, Ghana; d Department of Medicine, School of Medicine, University of Health and Allied Sciences (UHAS), Ho, Ghana; e Department of Speech, Language & Hearing Sciences, School of Allied Health Sciences, University of Health and Allied Sciences (UHAS), Ho, Ghana

**Keywords:** Esophageal perforation, Tilapia fish bone, Minimally invasive, Ultrasound, x-ray, Case report

## Abstract

**Introduction:**

Esophageal perforation (EP) is a rare, severe and challenging surgical emergency which can be caused by several factors. This report presents the case of a patient with EP caused by ingestion of a Tilapia fish bone.

**Presentation of case:**

A 41-year-old male patient presented with a six-day history of painful right-sided neck swelling, associated with a progressive dysphagia, regurgitation, fever and chills. Physical examination revealed an axillary temperature of 39 °C and a subcutaneous emphysema in the neck. Cervical spine x-ray and ultrasound scan revealed a neck foreign body with abscess. The case was managed by a right cervicotomy.

**Discussion:**

Despite over 20-years of clinical experience in Ghana, this was the first case of EP caused by a 2 cm long Tilapia fish bone, to be treated by the authors. This is significant because tilapia consumption is very popular in Ghana and it would be predicted that such cases would be more common. It is suggested that such cases do occur more frequently but are not reported to hospitals due to cultural-spiritual beliefs.

**Conclusion:**

Ingestion of Tilapia fish bones can cause significant damage to the esophagus and Tilapia should be consumed with care. Ultrasound is a very useful tool with high diagnostic accuracy for EP. Further studies are needed to establish the prevalence of EP from fish bone ingestion in Ghana and the factors accounting for the mismatch between the high consumption of Tilapia across the population and the low occurrence of patients presenting with EP from fish bone ingestion.

## Highlights

• Ingestion of tilapia fish bones can be cause esophageal perforation.
• Ultrasound is a very useful tool for diagnosing esophageal perforation.
• Further studies are needed to establish the prevalence of esophageal perforation in Ghana.

## 1 Introduction

Esophageal perforation is a rare, severe and challenging surgical emergency that can be caused by several factors. These factors include endoscopic examinations, surgical procedures, placement of tubes and intubation (i.e. iatrogenic causes) and non-iatrogenic causes such as penetrating wounds, thoracic trauma, swallowing foreign bodies and spontaneous rupture (i.e. Boerhaave syndrome) [1]. Esophageal perforation is associated with high mortality – 10–25% – when therapy is initiated within 24 h of perforation and 40–60% when the treatment is delayed [2]. The reason for higher mortality when treatment for esophageal perforation is delayed is due to the unique anatomical configuration and location of the esophagus, which allows bacteria and digestive enzymes easy access to the mediastinum, leading to the development of severe mediastinitis, emphysema, sepsis, and multiple organ dysfunction syndromes [3]. Consensus regarding the appropriate management of esophageal perforation is lacking. However, there is evidence to show that prompt diagnosis and effective treatment, such as complex open surgeries for closure or diversion of the esophagus with drainage of the mediastinum, are crucial in the management of esophageal perforation. This report presents the case of a patient with an esophageal perforation caused by swallowing a foreign body – a 2 in. long Tilapia fish bone, treated miniinvasively with cervicotomy. This case report has been reported using the SCARE checklist [4].

## 2 Presentation of case

**Fig. 1:**
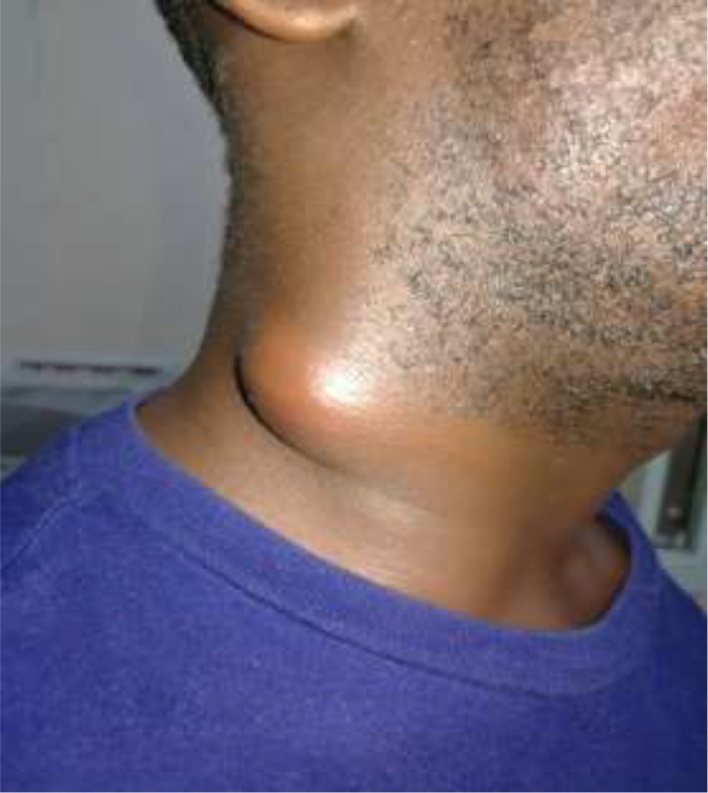
Patient with right-sided neck swelling.

**Fig. 2:**
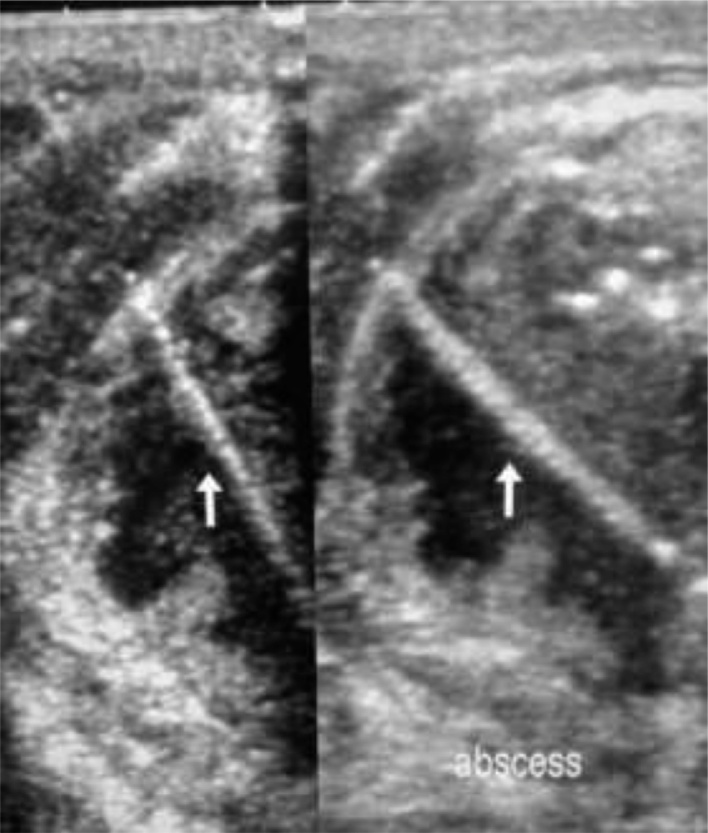
A USG scan showing neck with neck abscess caused by a 2 cm Tilapia fish bone (white arrowed).

A 41-year-old male patient with no history of hypertension, diabetes mellitus (DM), seizures, visual impairment, dysuria, surgery, hemotransfusion and asthma was referred to our hospital. The patient presented with a six-day history of painful right-sided neck swelling (Fig. 1), associated with a progressive dysphagia, regurgitation, fever and chills. Physical examination revealed an axillary temperature of 39 °C and a subcutaneous emphysema in the neck. The rest of his vital signs were within normal limits. The patient did not complain of dyspnea, coughing or choking on feeding. Cervical spine x-ray revealed a foreign body within the soft tissues. This was confirmed on an ultrasound (USG) scan which showed a hyperechoic, long and thin foreign body measuring 2 cm located 1.1 cm distance from the skin (white arrowed in Fig. 2). Wall thickening and a collection of dirty fluid was identified using a doppler signal. The USG scan confirmed the presence of a foreign body with abscess located on the right side of the neck. The surgical team requested a fluoroscopic radiological investigation using gastro-graffin or barium swallow, to make a diagnostic comparison with the USG report. Due to the absence of gastro-graffin or barium in our hospital, the fluoroscopic radiological investigation could not be performed. Using the clinical and imaging evidence from the cervical spine x-ray and the USG report, a diagnosis of esophageal perforation was made, and surgical intervention was conducted shortly after.

**Fig. 3:**
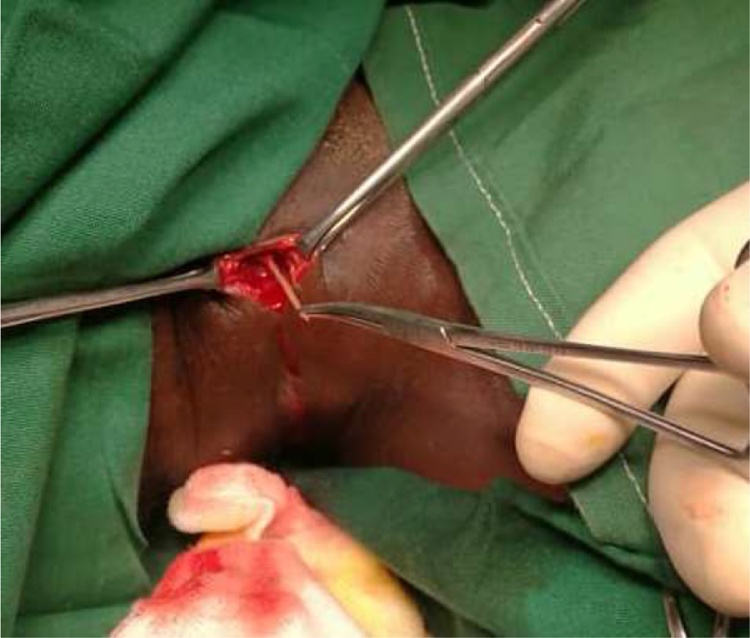
2 cm foreign body extraction through right cervicotomy.

Prior to the surgery, the patient signed the operation consent form per the hospital’s protocol. The patient was given general anaesthesia with spontaneous ventilation. The patient’s vitals prior to surgery were as follows: temperature 36.7 °C, blood pressure 120/90 mmHg, pulse rate 93 bpm, and respiratory rate 18 breaths per minute. The esophagus was accessed through a right cervicotomy with an incision of 2.5 cm midway between the anterior border of the sternocleidomastoid muscle. A 2 cm foreign body was extracted (Fig. 3) and 150 ml of pus was toileted and drained. A Penrose drain was secured to the skin with a simple suture using Nylon 3.0. After surgery, the patient was transferred to the ward and placed on antibiotics (cefuroxime 750 mg, metronidazole 500 mg administered intravenously within the first 48 h, and then subsequently metronidazole 400 mg tabs), anti-inflammatory and analgesic (paracetamol 1 g suppository). The patient made a favourable recovery, with frequent neck examinations and swallowing motility. After 24 h, the patient was able to swallow without difficulty, and without pain, and had no local and/or functional signs or symptoms. On post-operative day five (POD 5), there was no discharge from the wound and the patient was discharged home with an appointment for a review a week later. The patient reported to the hospital on the day of the appointment and the wound was dressed. The patient reported being satisfied with the treatment that he had received.

## 3 Discussion

This is a case report of a very rare esophageal perforation caused by the ingestion of a Tilapia fish bone. It was treated successfully using a minimally invasive approach which resulted in full patient recovery. This recovery can be attributed to prompt diagnosis and initiation of effective treatment. Studies report that esophageal perforation can be fatal if diagnosis and treatment are delayed [5,6]. In the current case, it was possible to quickly diagnose esophageal perforation because the patient presented with recognised signs and symptoms such as subcutaneous emphysema, which then precipitated the request for radiological examination. The patients’ recovery was also helped by the fact that the hospital in question was equipped with radiological services (conventional x-ray machine and USG) which aided in prompt diagnosis. It is a common phenomenon that many health facilities in Ghana are not equipped with radiological equipment such as conventional x-ray machines and USG. This is particularly common in rural areas. This case report supports evidence by Cavelier et al. [6] that USG is an excellent radiological modality with high sensitivity and specificity to esophageal perforation. However, if possible, fluoroscopic radiological investigation using gastro-graffin or barium swallow should be conducted before and after an esophageal perforation operation, to aid diagnosis and to also assess the success of the operation.

The case reported in this article was significant because it was the first time in over 20-years of clinical practice in Ghana, that the authors treated an esophageal perforation caused by a 2 cm long Tilapia fish bone. This is significant because Tilapia consumption is very popular in Ghana and it would be predicted that such cases would feature more often on hospital caseloads. The relative lack of such cases could mean that such incidents are indeed rare or are not being reported to hospitals. The authors suggest that the latter could be the main reason. Specifically, that this non-reporting could be attributed to the cultural beliefs of Ghanaians and a preference for using traditional medicine or consulting spiritual/religious figures for treatment. Cultural beliefs encompass the role of spirituality in health; health-related literacy and how health is prioritised in peoples’ lives. Ghanaian views of health may contrast with Western views and influence behaviour.

In Ghana, healthcare choices are often underpinned by spiritual and traditional beliefs [7,8]. The strong reliance on spiritual/traditional leaders for guidance and healing may lead to under-utilisation of essential and life-saving medication. This is because spiritual leaders are deemed by some as superior to qualified health professionals because their advice and/or suggestions are believed to come from God. Traditional medicine and spiritual practitioners deal with a wide variety of ailments. Common practices in Ghana include the use of herbal remedies, praying, and visiting/staying with a spiritual healer at a prayer camp in preference to hospital-based care. Those who seek help from both spiritual leaders and hospitals may find conflict between what each prescribes. For example, a spiritual leader might inform a patient that the cause of his illness is an evil spirit and that drugs and/or hospital care will not heal him. Thus, the patient may choose to not take advantage of available hospital services. It is possible that others choose traditional healers first and hospital care as a last resort. As traditional medicine has been found to be easily accessible and effective, it is possible that cases of esophageal perforation due to the ingestion of tilapia fish bone may have been successfully treated using these methods.

Across Ghana there is the need to improve healthcare education. Evidence indicates that many of the population, especially in the rural areas, have poor health literacy [9]. Access to basic health and sanitation education in rural Ghana is limited. Unlike many western countries where a proactive, preventative approach to healthcare is taken, incorporating the latest scientific advances, in Ghana a reactive approach is the norm [8]. Promotion of healthy habits is poor. Steps are not taken to prevent illness, and until physical symptoms appear, people may not realise they are sick. Appropriate facilities such as running water and toilets are also lacking. This contributes to the absence of basic hygiene practices such as hand washing with soap before eating, and after toileting. Habits such as open defecation are also widespread and are formed from an early age [[10], [11], [12], [13]]. When illnesses arise, there can be a failure to link symptoms to these everyday practices. For example, in relation to the topic of this paper, if a rural-based Ghanaian presented with dizziness, dyspnea, coughing, dysphagia, and swelling on the neck, it would unlikely be associated with an esophageal perforation. It would be more likely that these symptoms would be connected to a spiritual cause rather than diet. This would lead to a spiritual solution being sought instead of a medical one.

## 4 Conclusion

Ingestion of Tilapia fish bones can cause significant damage to the esophagus and be fatal if prompt diagnosis and treatment are not received. Ultrasound is a very useful tool with high diagnostic accuracy in relation to esophageal perforation. Further studies are needed to establish the prevalence of esophageal perforation in Ghana and understand the role of factors such as spiritual-cultural beliefs and poor health education in accounting for the mismatch between the high consumption of Tilapia across the population, and the low occurrence of patients presenting at hospitals with esophageal perforation from fish bone ingestion.

## Sources of funding

None. There were no sources of funding.

## Ethical approval

Ethical approval has been exempted by our institution.

## Consent

Written informed consent was obtained from the patient prior to the publication of this case report and its accompanying images.

## Declaration of Competing Interest

The authors declare that there is no conflict of interest or financial ties regarding the publication of this case report. This research did not receive any specific grant from funding agencies in the public, commercial, or not-for-profit sectors.

## Registration of research studies

NA.

## Guarantor

Seth Kwadjo Angmorterh accepts full responsibility for the article.

## Provenance and peer review

Not commissioned, externally peer reviewed.

## CRediT authorship contribution statement

**Radisnay Guzman Lambert:** Investigation, Data curation, Methodology, Writing - review & editing. **Seth Kwadjo Angmorterh:** Writing - review & editing, Writing - original draft, Methodology, Data curation, Investigation. **Manuel Betancourt Benjamin:** Investigation, Data curation, Writing - review & editing. **Mariuska Rodriguez Gonzalez:** Investigation, Data curation, Methodology, Writing - review & editing. **Sonia Aboagye:** Writing - review & editing. **Eric Kwasi Ofori:** Validation, Supervision, Writing - review & editing, Investigation.
